# Vibrational stability of a cryocooled horizontal double-crystal monochromator

**DOI:** 10.1107/S1600577516009243

**Published:** 2016-07-22

**Authors:** Paw Kristiansen, Ulf Johansson, Thomas Ursby, Brian Norsk Jensen

**Affiliations:** a FMB Oxford Ltd, Unit 1 Ferry Mills, Oxford OX2 0ES, UK; b MAX IV Laboratory, Lund University, Box 118, SE-221 00 Lund, Sweden

**Keywords:** horizontal double-crystal monochromator, vibrational measurements, vibrational performance, monochromator cryocooling

## Abstract

The vibrational stability of a horizontally deflecting double-crystal monochromator (HDCM) is investigated. Inherently a HDCM will preserve the vertical beam stability better than a ‘normal’ vertical double-crystal monochromator as the vibrations of a HDCM will almost exclusively affect the horizontal stability. Here both the relative pitch vibration between the first and second crystal and the absolute pitch vibration of the second crystal are measured. All reported measurements are obtained under active cooling by means of flowing liquid nitrogen (LN_2_). It is found that it is favorable to circulate the LN_2_ at high pressures and low flow rates (up to 5.9 bar and down to 3 l min^−1^ is tested) to attain low vibrations. An absolute pitch stability of the second crystal of 18 nrad RMS, 2–2500 Hz, and a relative pitch stability between the two crystals of 25 nrad RMS, 1–2500 Hz, is obtained under cryocooling conditions that allow for 1516 W to be adsorbed by the LN_2_ before it vaporizes.

## Abstract 2

The 25 nrad RMS, 1–2500 Hz, vibrational performance of a horizontal double-crystal monochromator is presented.

## 1. Introduction   

As synchrotron storage rings are approaching diffraction-limited performance (Eriksson *et al.*, 2014 ▸) it becomes crucial that the beamline optics are capable of preserving the brightness and coherence of the produced X-rays. This in turn means that the mechanical systems supporting the optics must be exceedingly stable. At the heart of most beamlines is a monochromator, and for X-ray energies from a few keV to ∼100 keV the most common monochromator is still a vertical double-crystal monochromator (DCM), where the monochromated beam maintains the direction of the incoming white beam with a vertical offset (Golovchenko *et al.*, 1981 ▸).

In liquid-cooled DCMs the flowing liquid will cause the first and second crystals to vibrate relative to each other. This relative pitch vibration between the two crystals is the main concern for DCMs deployed at beamlines requiring high beam stability as the relative vibration will cause the monochromated beam to lose its parallel direction relative to the incident beam with an amount corresponding to twice that of the relative vibration.

To best preserve the vertical beam emittance of the MAX IV storage ring (Leemann *et al.*, 2009 ▸) the DCM can be arranged such that the Bragg rotation offset is performed horizontally, which will virtually omit vertical beam distortions. In this article we report on the vibrational stability, both the relative pitch between the two crystals and the absolute Bragg stability of the crystal cage, of two identical horizontal double-crystal monochromators (HDCMs) that will be deployed on the NanoMAX (Johansson *et al.*, 2013 ▸) and BioMAX (Thunnissen *et al.*, 2013 ▸) beamlines at the MAX IV synchrotron in Lund, Sweden.

## 2. Experimental setup   

The measurements of the relative and absolute pitch vibrations were performed at the factory site of FMB Oxford in Oxford, UK. The measurements made using the vibrometer (see §2.4) were performed at the MAX IV Laboratory in Lund, Sweden, on the BioMAX beamline.

### 2.1. Motions of the HDCM   

**Figure 1:**
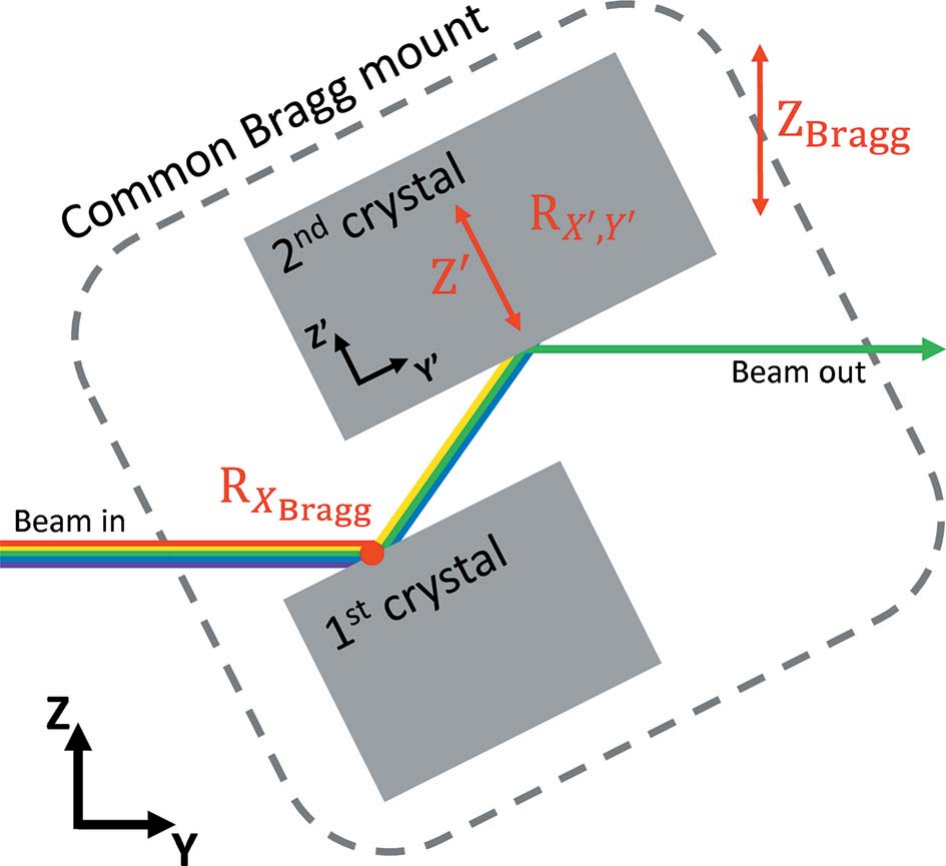
Schematic presentation of the HDCM’s degrees of freedom (not to scale). The white, rainbow-colored, beam enters from the left and the monochromatic beam exits at the right. Red letters indicate motion. The Bragg rotation axis is indicated by a red dot on the surface of the first crystal.

The investigated HDCM is highly versatile allowing the crystal motions listed in Table 1 ▸ and outlined in Fig. 1 ▸. All motions are encoded with Renishaw readheads. The HDCM is fitted with a pair of Si(111) crystals and can deliver a monochromatic beam from 5 to 30 keV with a nominal outboard horizontal offset of 10 mm. The crystals are mounted on a common rotational Bragg shaft ensuring that they rotate alike when the Bragg angle is changed (see Fig. 1 ▸).

#### 2.1.1. Bragg rotation   

**Figure 2:**
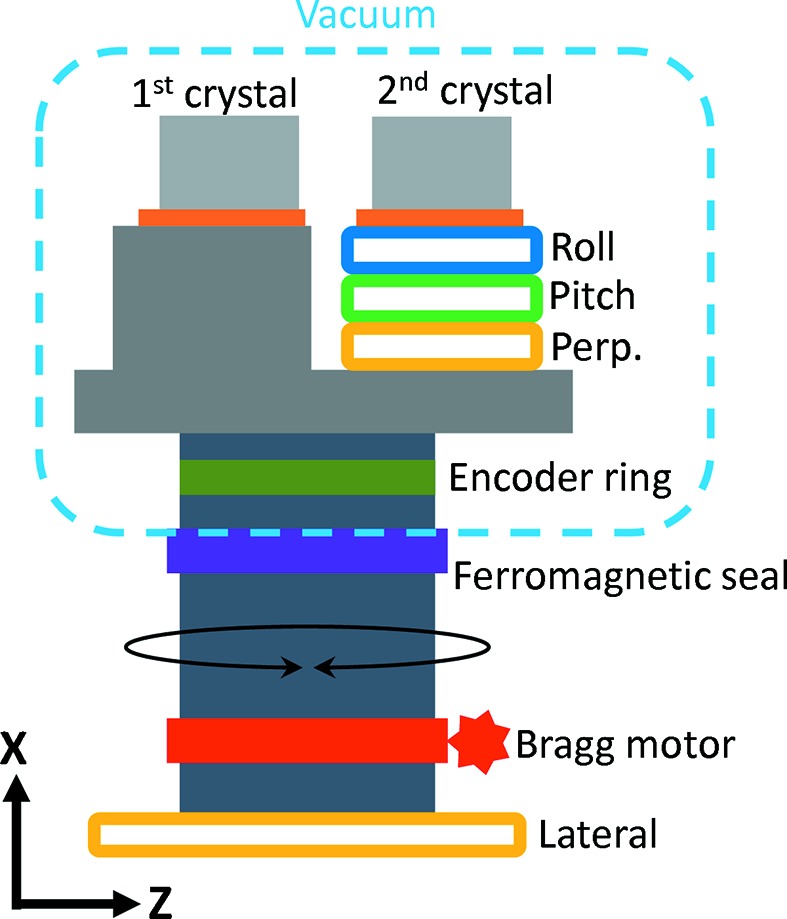
Schematic presentation of the Bragg and crystal cage assembly (not to scale). The cooling setup is not shown.

The Bragg rotation is achieved by a Phytron stepper motor with a harmonic drive gear both placed in air with a ferrofluidic seal (see Fig. 2 ▸). The maximum Bragg rotation speed is 1° s^−1^ and the measured resolution is 30 nrad. The Bragg encoder is a Renishaw Tonic that is located in vacuum.

#### 2.1.2. Cryocooling and vacuum   

**Figure 3:**
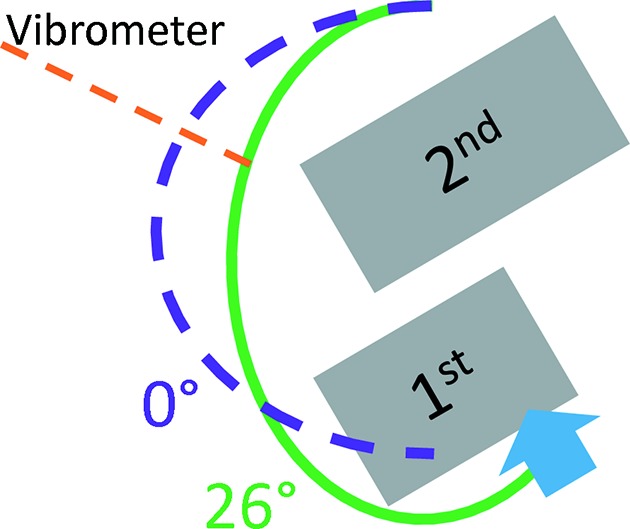
Schematic presentation of the in-vacuum cryoline support mechanism that bends with the Bragg angle (not to scale). The purple and green lines shown the cryolines at Bragg angles of 0° and 26°. The orange line shows where the vibrometer measurements were taken (see §2.4).

Both the first and second crystals are cryogenically cooled: the first crystal is dual side cooled by oxygen-free high-thermal-conductivity (OFHC) Cu blocks through which liquid nitrogen (LN_2_) is flowing. The LN_2_ was circulated by a FMB Oxford series D++ cryocooler (FMB Oxford Ltd). The cooling is designed for the first crystal to absorb 110 W with a peak density of 80.8 W mm^−2^ when the crystal is orthogonal to the beam, which at 5 keV corresponds to 32 W mm^−2^. The second crystal is also dual side cooled by OFHC Cu blocks, though these blocks are cooled by Cu braid connections to the cooling Cu blocks of the first crystal. The LN_2_ is supplied to the first crystal by means of internally braided (to reduce flow vibrations) flexible steel hoses that are supported by a sheet steel plate that is pulled into a spiral shape as the Bragg angle is increased (see Fig. 3 ▸).

The pressure of the circulating LN_2_ is proportional integral derivative (PID) controlled by an immersion heater placed in a buffer volume of LN_2_ that is in direct connection with the circulating LN_2_. The PID feedback pressure is measured in the buffer and, as the LN_2_ pump is downstream of the buffer, the pressures experienced at the Bragg crystals, half way through the high-pressure circuit, will be slightly higher than the controlled pressure in the buffer as the dynamic pressure of the pumping is to be added to the buffer pressure.

Prior to cooling, the HDCM pressure was brought down to ∼1 × 10^−5^ mbar by means of a turbo pump. At this pressure LN_2_ is led into the cooling circuit and the system was left to thermally stabilize for ∼8 h. During measurements the HDCM was sealed off without any vacuum pumping. Once installed at the beamlines the HDCM will be pumped by an ion pump that will have no vibrational impact.

### 2.2. Relative pitch vibrations   

**Figure 4:**
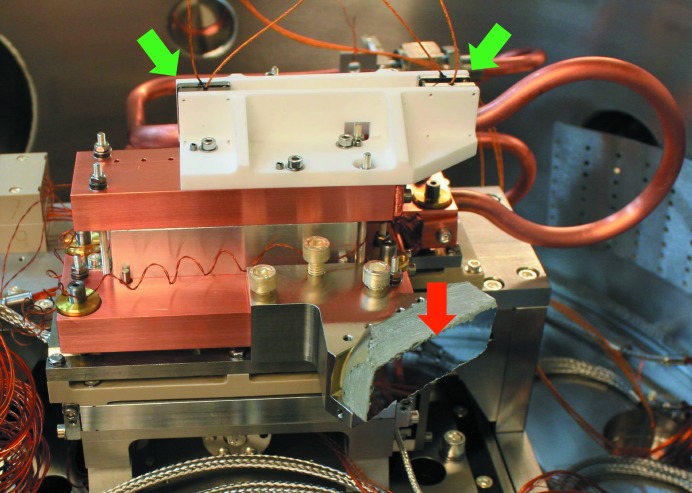
Photograph of the back of the second Al dummy crystal. The capacitive sensor sets, used for relative pitch vibration measurements, are marked with green arrows. The mirror, used for absolute pitch vibration measurements of the second crystal, is marked with a red arrow.

In order to measure the relative pitch vibration between the two Bragg crystals two sets of Queensgate capacitive distance sensors, of the NXC Al series, were used. The distance sensors were controlled by two NS2000-S units, also from Queensgate, configured in a master/slave relation. The capacitive distance sensors are capable of measuring with an accuracy of 0.1 nm with a bandwidth of 5 kHz (Nanopositioning. Nanosensors, nx series). The full bandwidth of 5 kHz was used for the measurements and they were made over 1 s. Note that 5 kHz is the measurement rate and thus physical vibrations up to 2.5 kHz will be detected. The cap sensors are mounted rigidly to the crystals on ceramic brackets (see Fig. 4 ▸) where the capacitive distance sensors are marked with green arrows. This arrangement allows for a direct measurement of the relative pitch vibration over the full Bragg range. For these measurements Al dummy crystals were used.

### 2.3. Absolute pitch vibrations   

An interferometric quarter-wave-plate setup like that described by Kristiansen *et al.* (2015 ▸) was used to measure the absolute pitch vibration of the second crystal (see Fig. 4 ▸, where the target mirror is marked with a red arrow). The interferometer laser used was model XL-80 from Renishaw. The measurement bandwidth used was 5 kHz and 2^13^ data points were recorded.

As the interferometer requires an orthogonal view of the target mirror all absolute measurements were made at a Bragg angle of 9° since this was the only angle offering a flange suitable for a view port. Part of the laser path is in air which brings artificial distortions in the low-frequency range. These distortions were removed by subtracting a polynomial fit from the raw data before calculating the RMS value and the fast Fourier transform (FFT) spectrum. This effectively removes vibrations below 2 Hz from the data.

### 2.4. Cryoline support vibrations   

In order to measure the vibration of the cryoline support mechanism (see Fig. 3 ▸) a laser Doppler vibrometer from Polytec, model OFV-534 run by a OFV-5000 vibrometer controller, was deployed. The measurements were recorded with a 1 kHz bandwidth for 5 min. The measurements were taken through a viewport of the cryoline support. The position of the measurement changes with Bragg angle (see Fig. 3 ▸).

The point of these measurements is to determine to what extent the vibrations of the cryoline support mechanism migrates to the crystals, why both the cryoline support mechanism and the second crystal vibration were measured with the vibrometer.

## 3. Results   

**Figure 5:**
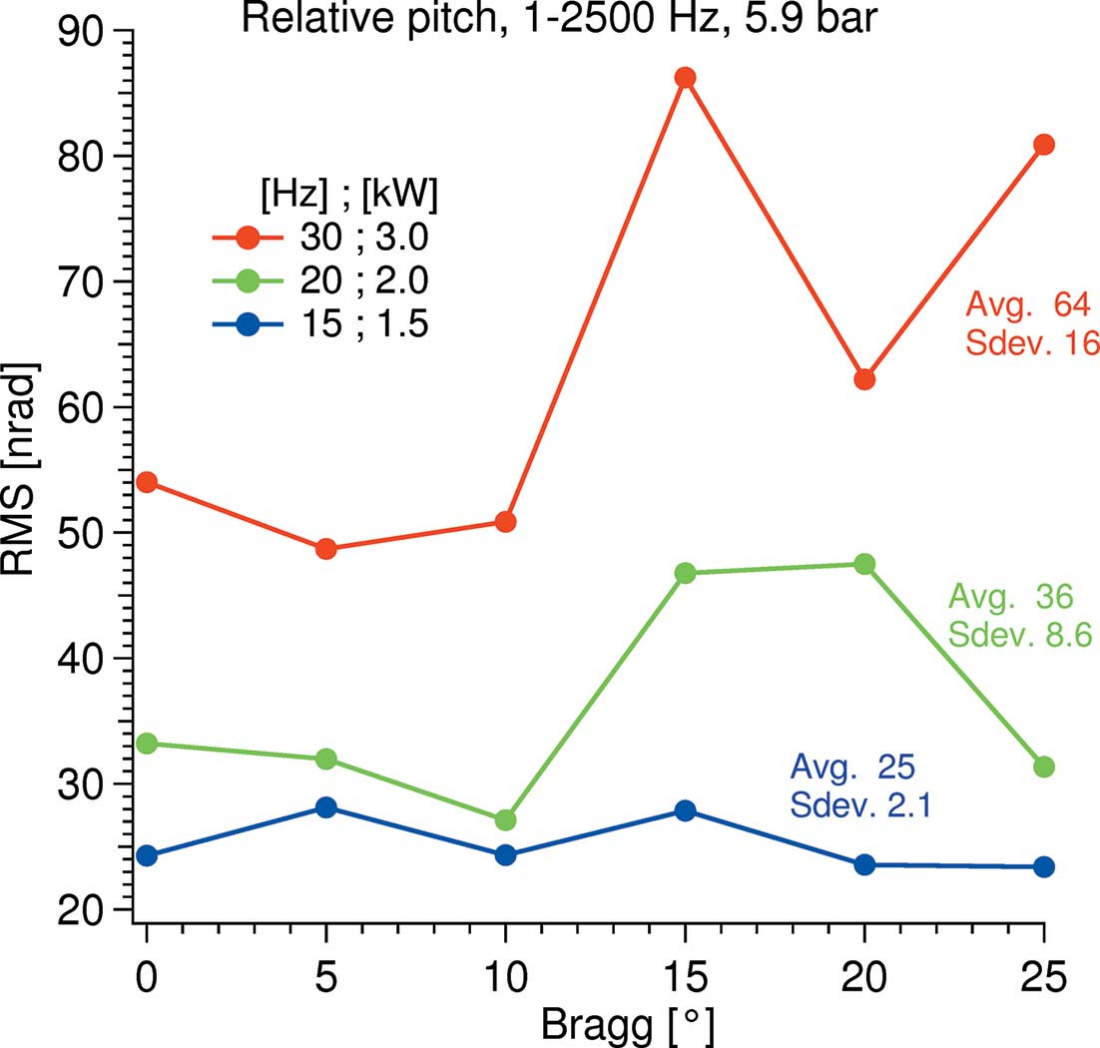
Relative pitch vibration over the Bragg range with a cryocooler pressure setting of 5.9 bar and various pump speeds. The legend shows the calculated potential cooling power at the three pump speeds. The ‘Avg.’ number is the average of the trace and the ‘Sdev.’ number is its standard deviation.

The headline results are given in Table 2 ▸. The listed relative pitch is the average of six equally spaced measurements over the full Bragg range which has a standard deviation of 2.1 nrad (see Fig. 5 ▸). The absolute pitch vibration of the second crystal is recorded at a Bragg angle of 9°. Both vibrations were recorded with a pump speed of 15 Hz, 3 l min^−1^, and a LN_2_ pressure setting of 5.9 bar amounting to the listed potential cooling power (CP; see §4.1 for details of the CP).

## 4. Discussion   

### 4.1. Relative pitch vibration   

In Fig. 5 ▸ the relative pitch vibration over the Bragg range is displayed with a 5.9 bar LN_2_ pressure and three different pump speeds corresponding to 3, 4 and 6 l min^−1^. The higher pumping rates have relatively large standard deviations, which is assumed to be due to the in-vacuum cryoline arrangement that at high flows is more susceptible to oscillate under certain combinations of conditions: the in-vacuum cryolines are supported by a sheet metal that bends as the Bragg angle changes (see Fig. 3 ▸), so the spring constant of the arrangement is dependent on LN_2_ flow, LN_2_ pressure and Bragg angle.

To compare vibrational levels across different settings of the cryocooler the potential CP of the settings can be calculated as 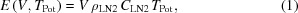
where *E* is the potential available CP (before the LN_2_ starts to form gaseous N_2_), *V* is the flow rate of the LN_2_, 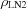 = 803 kg m^−3^ is the density of LN_2_, 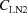 = 2042 J (kg K)^−1^ is the heat capacity of LN_2_ and 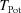 is the difference in temperature between the LN_2_ sacrificial cooling bath of the cryocooler, 77 K, and the boiling temperature of the circulating LN_2_ at the sat pressure, *e.g.* the LN_2_ pump pumping rate is 1 l min^−1^ per 5 Hz and 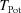 at a sat pressure of 5.9 bar is 18.5 K which then equates to *E*(3 l min^−1^, 18.5 K) = 1516 W. The used 1/2-inch cryolines connecting the cryocooler and the HDCM will typically only consume 2 W m^−1^ (AS Scientific Products) and the Cu cooling blocks are thermally isolated from the bulk of the HDCM by PEEK (polyether ether ketone) so the calculated figure will not be too far off the cooling power actually available for the crystals when equilibrium is reached. Note, though, that this calculated potential cooling power by no means reflects a suggested maximum heat absorption into the crystals as this would severely deform them (Rutishauser *et al.*, 2013 ▸). The calculated potential cooling power serves as a means to compare different cryocooler settings and it shows whether the used setting can deliver enough cooling for the intended crystal heat absorption, here 110 W.

**Figure 6:**
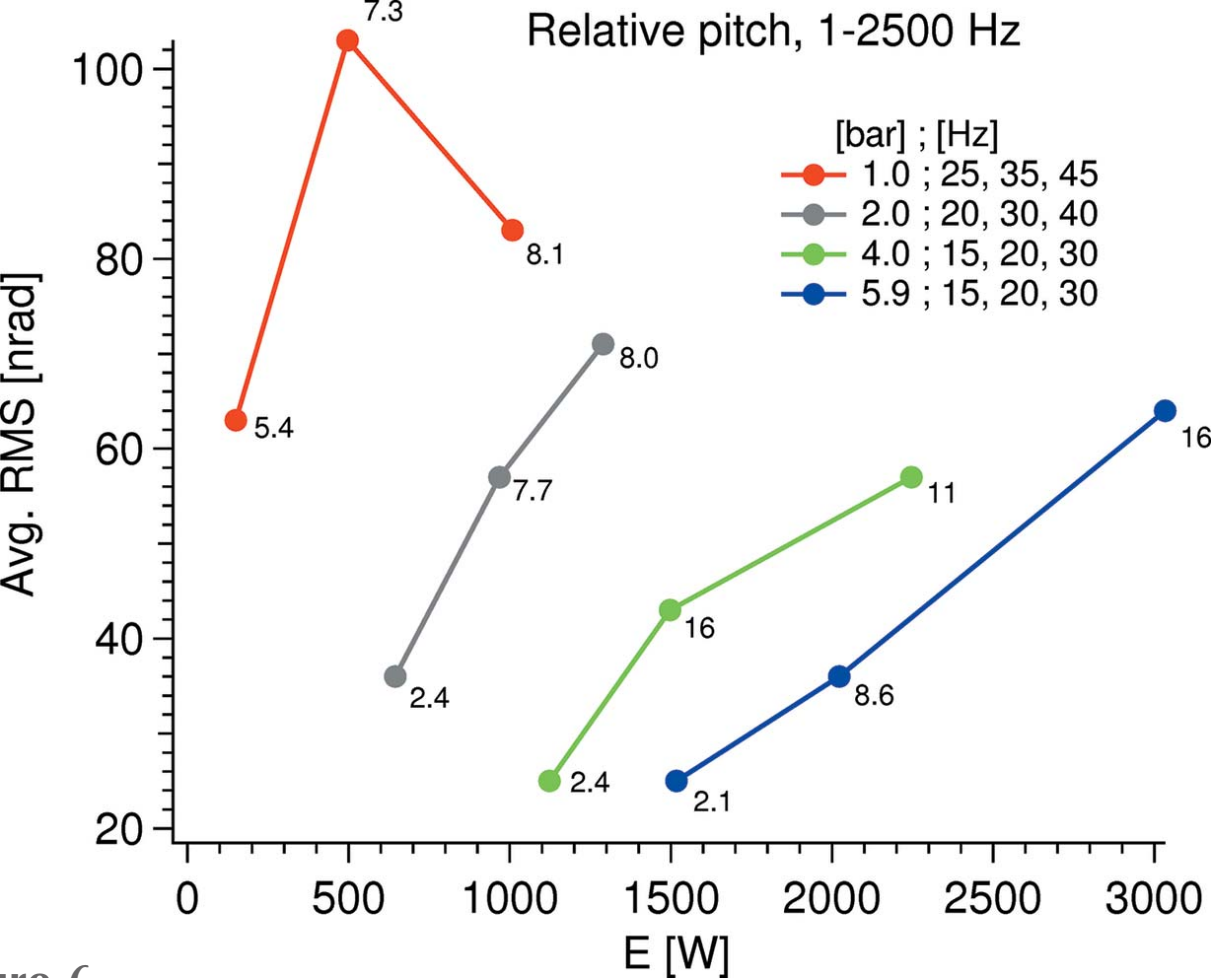
Relative pitch vibration at different potential cooling powers. In the legend, the first number in the label is the sat pressure of the cryocooler and the subsequent three are the pump speeds. The number next to the data points is the standard deviation of the six Bragg angles used to calculate the displayed average value.

In Fig. 6 ▸ the averaged RMS value over six equally spaced angles covering the Bragg range against the potential cooling power is shown. From this it is evident that running at high pressures, which will stiffen up the in-vacuum cryoline arrangement, and low pump speeds, which will promote a laminar flow, is favorable for minimum vibration. Also, the lower pump speed promotes less fluctuations over the Bragg range as shown by the low standard deviations.

**Figure 7:**
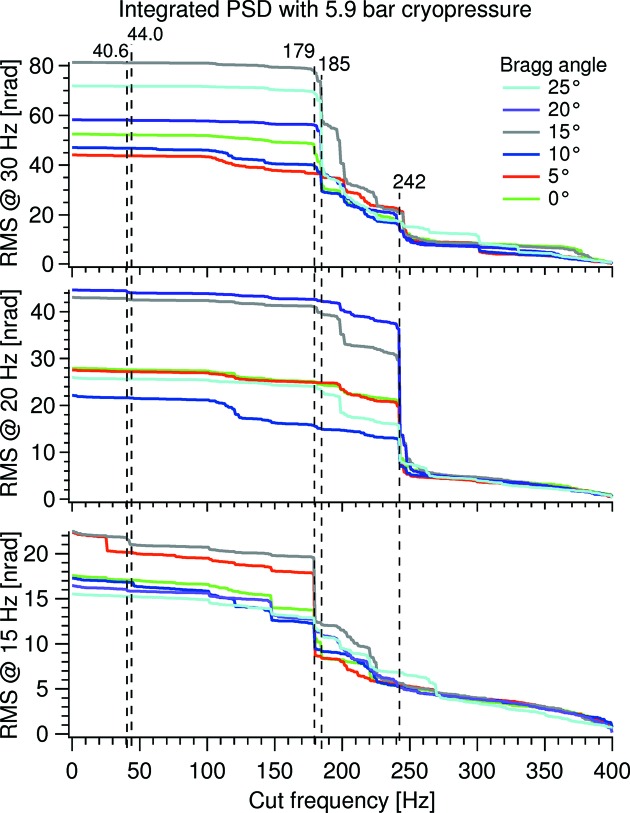
Integrated PSD from cut frequency to 400 Hz of the relative pitch vibration with the sat cryocooler pressure at 5.9 bar and a pump speed of 15, 20 and 30 Hz (bottom, middle and top). The shown data are spectral information on the RMS data points of Fig. 5 ▸.

Fig. 7 ▸ shows the integrated power spectrum density (PSD) of the RMS values from Fig. 5 ▸, with the integration starting at 400 Hz [see Kochanczyk *et al.* (2012 ▸) for details on integrated PSDs]. These spectra could suggest that the LN_2_ pump speed, 15, 20 and 30 Hz, equating to 3, 4 and 6 l min^−1^, respectively, is the predominant factor in the interplay between it and the varying stiffness of the in-vacuum cryoline support over the Bragg range (see Fig. 3 ▸) at a LN_2_ circulating pressure of 5.9 bar: each of the three conditions show major contributions at different frequencies, 179, 242 and 185 Hz at 15, 20 and 30 Hz LN_2_ pump speed, respectively. The level of dominance is dependent on the Bragg angle, but remains at the same frequency. This indicates a varying amplification with changing Bragg angle. A varying amplification is possibly due to a shift of frequency for nearby resonances.

The first point of interest in improving the vibrational performance of the investigated setup would thus be to improve the flow path of the LN_2_ to obtain a higher level of laminar flow.

The characteristics of Fig. 7 ▸, with one dominant frequency at a given flow, are also seen at lower sat LN_2_ pressures, but become increasingly less pronounced as the LN_2_ pressure goes down (data not shown).

### 4.2. Absolute pitch vibration   

**Figure 8:**
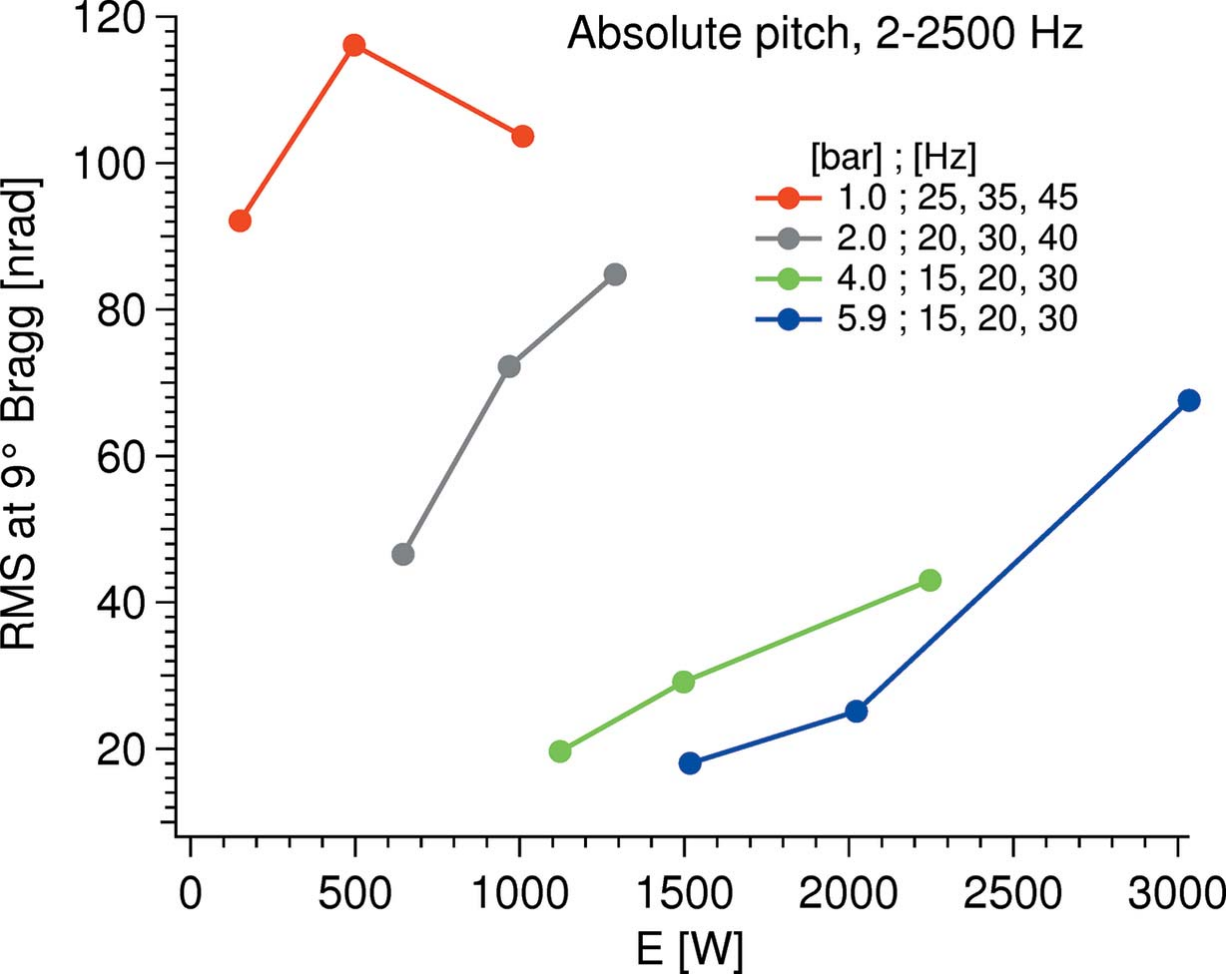
Absolute pitch vibrations at 9° Bragg at different potential cooling powers. In the legend, the first number in the label is the sat pressure of the cryocooler and the subsequent three are the pump speeds.

The RMS values of the absolute pitch vibrations of the second crystal at 9° Bragg are displayed in Fig. 8 ▸. Compared with the relative pitch vibrations in Fig. 6 ▸, it can be seen that the trend is the same but between the different pressures the vibrational levels are more spread in absolute terms. This sensitivity towards the cryocooler pump speed is most likely due to vibrations originating from the, presumingly, non-laminar flow in the internal cryolines which vibrate the entire crystal cage assembly dominantly as a rigid body. Even though the absolute vibration has about the same magnitude as the relative, the absolute vibration is of significantly less concern: an absolute vibration of 120 nrad RMS at 10.15°, corresponding to the *K*α Se fluorescence energy at 11.22 keV on the Si 111 crystals, leads to an energy jitter of 7 meV RMS off the first crystal and a beam offset change of 0.2 nm RMS.

### 4.3. Absolute vibration migration   

**Figure 9:**
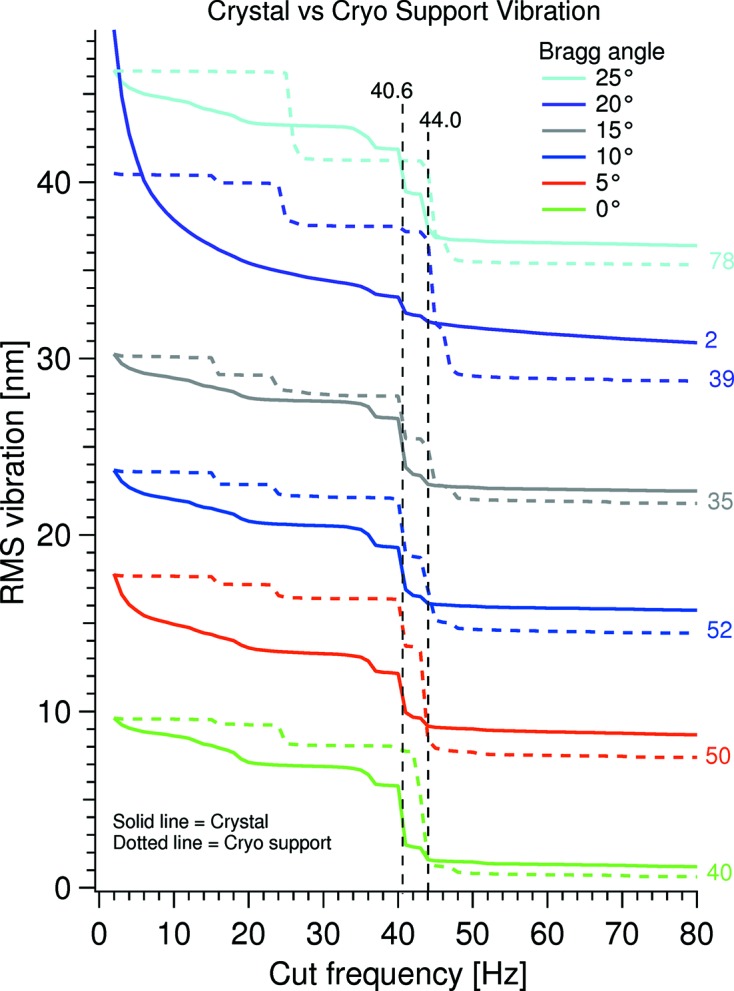
Integrated PSD from cut frequency to 350 Hz of the linear vibration of the second crystal *versus* the linear vibration of the cryoline support mechanism. The numbers to the right of the traces are the division factors used to norm the cryo support vibration to the crystal vibration at 2 Hz. The crystal vibration data are offset 7 nm between the Bragg angles. All the shown data were obtained using a cryo pump speed of 15 Hz and a flow pressure of 5900 mbar.

Fig. 9 ▸ shows the integrated PSD of the linear vibration of the second crystal *versus* the linear vibration of the cryoline support mechanism with the integration starting at 350 Hz. The crystal vibration traces are offset by 7 nm between them and the solid trace at 20° Bragg has been divided by 2. The linear vibration of the cryoline support mechanism has been normalized pairwise to the linear crystal vibration at 2 Hz. The relatively large division factor is a result of the crystal cage being directly supported on the HDCM granite base and the crystal cage assembly being considerably more rigid than the cryoline support mechanism.

It is seen that the linear crystal vibration above 40 Hz originates from the linear cryoline support mechanism vibration with two modes at 40.6 and 44.0 Hz. Faintly these modes are visible in the lower graph in Fig. 7 ▸ of the relative pitch vibration, but are nearly negligible compared with the main relative pitch vibration at 179 Hz. This suggests that vibration of the cryoline support mechanism is not the main source of the relative pitch vibration, but rather that this originates from crystal cage modes excited from LN_2_ flow in the rigidly mounted Cu flow tubes fixed to the crystal cage and the OFHC Cu cooling blocks.

### 4.4. Relative- and absolute-pitch vibration comparison   

**Figure 10:**
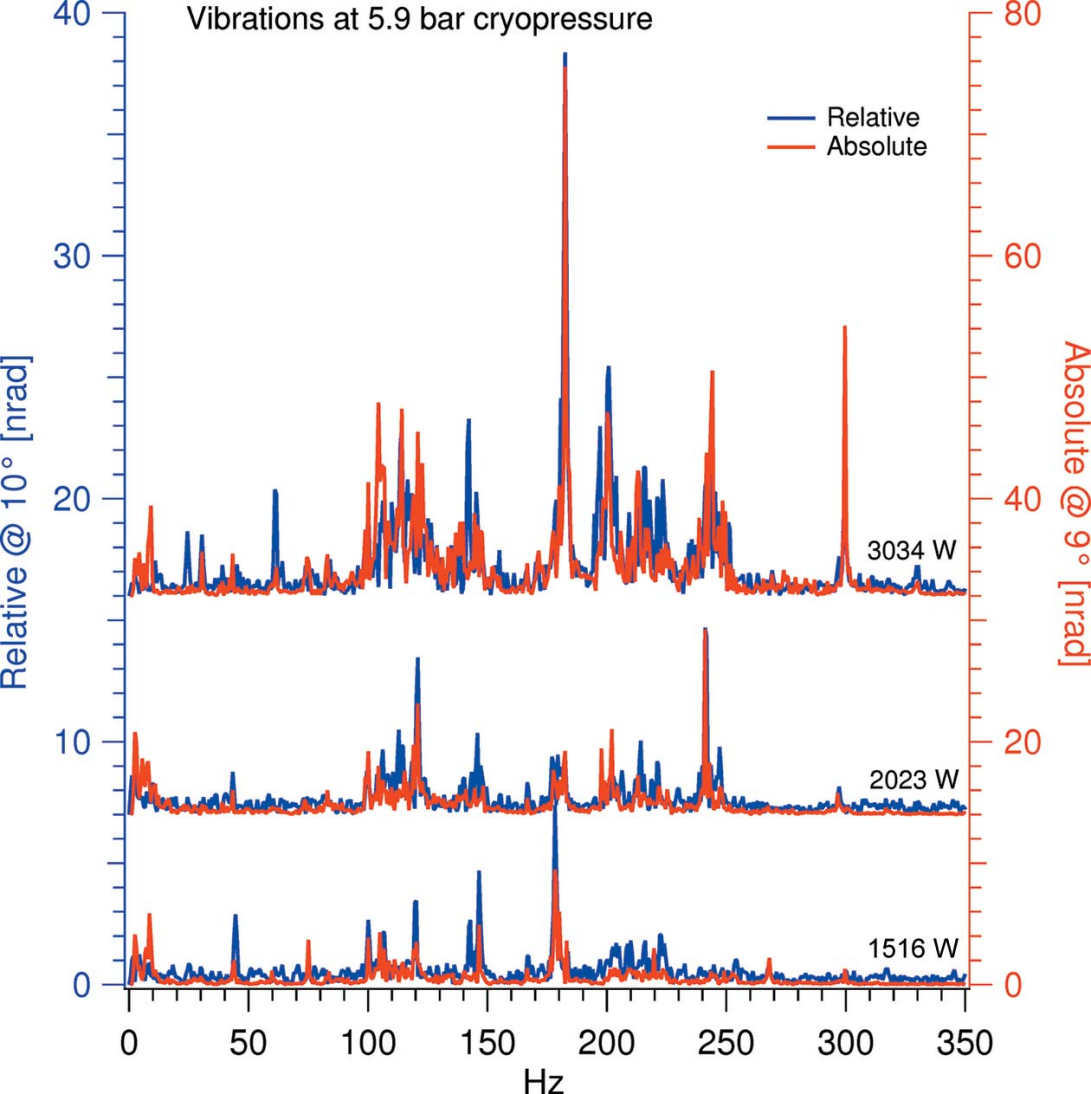
Vibration spectrum with a cryopressure of 5.9 bar and pumping speed of 15, 20 and 30 Hz. Note that the relative and absolute vibrations are on separate axes and that the four top spectra have been offset.

In Fig. 10 ▸ the relative and absolute vibration FFT spectra are compared under alike conditions, the relative data originating from a 10° Bragg angle and the absolute data from a 9° Bragg angle but otherwise pairwise the same. The three paired spectra are recorded with a potential cooling power of 1516, 2023 and 3034 W.

As the relative and absolute vibration largely shares peak frequencies, in all the pairs, it is a reasonable assumption that the source of the vibrations is non-laminar LN_2_ flow: the flow vibrations vibrate the crystal cage which causes a relative vibration as the internal crystal cage assembly is not fully rigid.

## 5. Conclusions   

The investigated HDCM is found to have a relative pitch vibration of 25 nrad RMS, in the 1–2500 Hz physical vibration band, when circulating the LN_2_ at 5.9 bar and 3 l min^−1^, corresponding to a potential cooling power of 1516 W. It is shown that it is vibrationally favorable to run the cryocooler at low pump speeds and high pressures. The main cause of the relative pitch vibration is suggested to be from crystal cage modes that are excited by the flow of LN_2_ in Cu tubes rigidly mounted to the crystal cage.

## Figures and Tables

**Table 1:** Motion ranges of the investigated HDCM

Motion	Range
Bragg rotation, *R*_*X*Bragg_	26°
Bragg mount lateral, *Z*_Bragg_	6 mm
Second crystal pitch, *R*_*X*′_	2°
Second crystal roll, *R*_*Y*′_	2°
Second crystal perpendicular, *Z*′	3 mm

**Table 2:** Measured relative and absolute vibrations

Vibration	Cooling power (W)	Frequency range (Hz)	RMS (nrad)
Relative pitch	1516	1–2500	25
Absolute pitch	1516	2–2500	18
